# MitoTEMPOL modulates mitophagy and histopathology of Wistar rat liver after streptozotocin injection

**DOI:** 10.22038/IJBMS.2022.65285.14375

**Published:** 2022-11

**Authors:** Rova Virgana, Julia Windi Gunadi, Nur Atik, Kwee Limdawati, Diana Krisanti Jasaputra, Roro Wahyudianingsih, Nadya Naurah Adrina Suardi, Ray Sebastian Soetadji, Hanna Goenawan, Ronny Lesmana, Arief Sjamsulaksan Kartasasmita

**Affiliations:** 1 Department of Ophthalmology, Universitas Padjadjaran, Bandung, West Java, Indonesia; 2 Cicendo National Eye Hospital, Bandung, West Java, Indonesia; 3 Department of Physiology, Faculty of Medicine, Maranatha Christian University, Bandung, West Java, Indonesia; 4 Biology Cell Division, Department of Biomedical Sciences, Faculty of Medicine, Universitas Padjadjaran, Bandung, West Java, Indonesia; 5 Department of Internal Medicine, Faculty of Medicine, Maranatha Christian University, Bandung, West Java, Indonesia; 6 Department of Pharmacology, Faculty of Medicine, Maranatha Christian University, Bandung, West Java, Indonesia; 7 Department of Pathology Anatomy, Faculty of Medicine, Maranatha Christian University, Bandung, West Java, Indonesia; 8 Faculty of Medicine, Maranatha Christian University, Bandung, West Java, Indonesia; 9 Physiology Division, Department of Biomedical Sciences, Faculty of Medicine, Universitas Padjadjaran, Bandung, West Java, Indonesia; 10 Physiology Molecular Laboratory, Biological Activity Division, Central Laboratory, Universitas Padjadjaran, Jatinangor, West Java, Indonesia; # These authors contributed eqully to this work

**Keywords:** Anti-oxidants, Lipid droplet, Mitochondrial dynamics, Mitophagy, Metabolic zone, Oxidative stress

## Abstract

**Objective(s):**

This study aims to explore the effect of mitoTEMPOL on histopathology, lipid droplet, and mitophagy gene expression of Wistar rat’s liver after injection of streptozotocin (STZ).

**Materials and Methods:**

Twenty male Wistar rats were divided into 4 groups: Control (n=5); 100 mg/kg BW/day mitoTEMPOL orally (n=5); 50 mg/kg BW STZ intraperitoneal injection (n=5); and mitoTEMPOL+STZ (n=5). STZ was given a single dose, while mitoTEMPOL was given for 5 weeks after 1 week of STZ injection. Histopathological appearance, lipid droplets, mitophagy, and autophagy gene expression were examined after the mitoTEMPOL treatment.

**Results:**

We found metabolic zone shifting that might be correlated with the liver activity of fatty acid oxidation in the STZ group, a decrease of lipid droplets in mitoTEMPOL and mitoTEMPOL + STZ compared with Control and STZ groups were found in this study. We also found significant changes in PINK1, Parkin, BNIP3, Mfn1, and LC3 gene expression, but no difference in Opa1, Fis1, Drp1, and p62 gene expression, suggesting a change of mitochondrial fusion rather than mitochondrial fission correlated with mitophagy.

**Conclusion:**

All this concluded that mitoTEMPOL could act as a modulator of mitophagy and metabolic function of the liver, thus amplifying its crucial role in preventing mitochondrial damage in the liver in the early onset of diabetes mellitus.

## Introduction

Diabetes mellitus is a serious disease that can cause complications in various organs of the body, thereby reducing the quality of life (1). Chronic hyperglycemia in diabetes causes glucotoxicity that might be correlated with mitochondrial dynamics (2). Glucotoxicity refers to excess carbohydrate intake in tissues that might be converted to triglycerides, free fatty acid, and free cholesterol, inducing steatosis in the liver (3). Recent studies have even shown that glucotoxicity can cause liver cell injury by inducing endoplasmic reticulum stress and hepatocyte cell death (3).

A chemical that is widely used for inducing diabetes type 1 and 2 in rodents is STZ (4-6). A single STZ injection causes partial destruction of the pancreas, providing a good model for the effect of glucotoxicity and lipotoxicity on mitochondrial oxidative dysfunction (4, 6). This single-dose STZ injection is less expensive and less time-consuming, compared with other models that induce diabetes (4). With its glucose moiety that is linked to β cells, STZ recognizes GLUT2 receptors in β cell plasma membranes, liver, and kidney (4, 7, 8). After injection, STZ is quickly metabolized by the liver and then excreted by the kidney, therefore its acute toxic effect on those organs could be neglected, then the effect of glucotoxicity on the liver could be extensively studied in this model (4, 9). 

Although liver weight is only 2-4% of total body weight, it has a high oxygen consumption, approximately 60% of the organismal mitochondrial ATP demand is covered by hepatocytes (10, 11). Mitochondria in hepatocytes is participating in lipid and glucose production, storage, and consumption, free radicals production, cell survival, and death (12, 13). Chronic hyperglycemia was proven to be the contributing factor that increases ROS production in mitochondria (14, 15). Overproduction of ROS leads to oxidative stress where the balance of ROS and antioxidants in the cells is disturbed (14). This condition might lead to mitochondrial dysfunction characterized by reduced mitochondrial biogenesis, numbers, activities, and altered membrane potential, further damaging other cell organelles, membranes, and proteins then finally inducing apoptosis (13, 16, 17). Therefore, maintaining homeostasis by recycling damaged organelles is needed to support mitochondrial dynamics, which is achieved by the work of autophagy, or more specifically mitochondrial autophagy (mitophagy) (17, 18).

Mitophagy is a process of maintaining homeostasis by eliminating damaged mitochondria (18). It could be induced through several pathways: PINK1/Parkin, BNIP/NIX, and FUNDC1, but the dominant pathway is PINK1/Parkin.(19-22) PINK1 is normally transported to the Inner Membrane Membrane (IMM) and then cleaved by the inner membrane protease PARL (Presenilins-associated rhomboid-like protein), but in mitochondrial dysfunction where there is a reduced membrane potential, PINK1 could not be transported into the IMM (22-24). This process makes the damaged mitochondria easily detected by autophagosomes, combined with Parkin recruitment that induces phosphorylation of Mfn1 and Mfn2 (22, 25, 26). PINK1/Parkin is activating mitophagy through indirect binding to autophagosomes, on the contrary, BNIP/NIX is activating mitophagy through indirect binding to autophagosomes (22, 25). These two pathways of mitophagy are induced by metabolic stress, oxidative stress, and fatty acid accumulation that eventually induced mitochondrial oxidative dysfunction (22, 25, 27).

Another way to improve mitochondrial oxidative dysfunction is by increasing antioxidants, especially mitochondria-targeted antioxidants (MTA). MTA could cross the mitochondrial phosphate bilayer and then eliminate excessive ROS at its central source (28). MitoTEMPOL is an MTA, derivative of TEMPOL (4-hydroxy-2,2,6,6- tetramethylpiperidine-1-oxy radical), which exhibits potent antioxidant properties. It contains the piperidine nitroxide (TEMPOL) and lipophilic cation triphenylphosphonium (TPP+) moiety. Tempol is a superoxide dismutase mimetic that dismutases superoxide in the catalytic cycle, whereas TPP is a membrane cation that accumulates several hundred-fold in the mitochondria because of the membrane potential. This combination creates mitochondrial targeting antioxidants that have effective superoxide scavenging abilities (29). Interestingly, MitoTEMPOL content has shown its ability to protect the liver from various injuries, such as liver-endotoxin injury, sepsis, hypertension, or colitis (30). Nevertheless, its molecular mechanism in the early stage of diabetes mellitus as a prevention strategy for complications in the liver is still unclear. This study aims to explore the effect of mitoTEMPOL on histopathology, lipid droplet, and mitophagy gene expression after the injection of single-dose STZ.

## Materials and Methods

***Animals***

All animal protocols of the experiment were approved by the Research Ethics Committee of the Faculty of Medicine, Universitas Kristen Maranatha (No 165/KEP/VII/2021). Twenty 8-week old male Wistar rats weighing 210±20 g (n=5/group) were divided into 4 groups: 1) Control, 2) MitoTEMPOL 100 mg/kg BW/day orally, 3) STZ intraperitoneal injection 50 mg/kg BW, and 4) STZ intraperitoneal injection 50 mg/kg BW and MitoTEMPOL 10 mg/kg BW/day orally. Male Wistar rats were purchased form Biofarma, Bandung, Indonesia. The rats were given a standard chow diet and were housed at room temperature with 12 hr of light and dark cycles every day. We conducted all procedures based on the use and care of laboratory guidelines. 

***MitoTEMPOL and STZ dose***

MitoTEMPOL was purchased from Sigma-Aldrich Co. (Saint Louis, Missouri, USA), and STZ was purchased from Cayman Chemical Co. (Ann Arbor, Michigan, USA). We used a dose of MitoTEMPOL, 100 mg/kg BW for each rat, per os, 5 times a week, for 5 weeks while STZ in DMSO and sterile MilliQ was given via peritoneal injection, 50 mg/kg BW single dose. The dose of MitoTEMPOL and STZ was based on previous studies (4, 31). After 5 weeks, animals were euthanized (32), and the livers were rapidly excised and weighed. Then the livers were taken, and two sets of experiments were conducted, one for histopathological examination and the other for RNA extraction continued with semiquantitative PCR of mitophagy gene expression.

***Hematoxylin and eosin (H&E staining)***

For histopathological examination, the liver was fixed immediately in 10% formalin after extraction. The specimens were processed for paraffin embedding followed by the preparation of 2-𝜇m thick sections. The sections were stained with hematoxylin and eosin (H&E) for light microscope examination (LEICA ICC50, Wetzlar, Germany) with 100x and 400x magnification. Images were captured using Leica Application Suite (LEICA, Wetzlar, Germany). Images were analyzed by an expert pathologist. We evaluated the histopathological changes using a scoring system that had been used in our previous study (33). The evaluation of the livers was divided into three categories: congestion/sinusoidal dilatation, cloudy swelling/injury, and inflammation. For scoring of liver congestion or sinusoidal dilatation score, sections were assessed based on liver zones, using this following scoring: 0=No congestion/sinusoidal dilatation; 1=Mild congestion or centrilobular (zone III) sinusoidal dilatation; 2=Moderate congestion or centrilobular (zone II) sinusoidal dilatation; and 3=Severe congestion or centrilobular (zone I) sinusoidal dilatation. For scoring of cloudy swelling/hepatic injury, we used the following scoring: 0=No cloudy swelling/hepatic injury; 1=Mild cloudy swelling/hepatic injury (zone III); 2=Moderate cloudy swelling/hepatic injury (zone II); 3=Severe cloudy swelling/hepatic injury (zone I). For scoring the inflammation severity, we used the following scoring: 0=No hepatic inflammation; 1=Mild hepatic inflammation or periportal inflammation; 2=Moderate hepatic inflammation or periportal and intraparenchymal inflammation; and 3=Severe hepatic inflammation or periportal and intraparenchymal inflammation with bridging necrosis (33). 

***Oil red O staining***

For lipid droplet quantification, we used oil red O staining. The oil red O was purchased from Sigma Aldrich Co. (Saint Louis, Missouri, USA). We used 0.5-gram oil red O dissolved in 100 ml isopropanol to make the oil red O stock stain, then we diluted 30 ml of the stock stain with 20 ml of distilled water to make the oil red O working solution. The specimens were processed for paraffin embedding followed by the preparation of 2-𝜇m thick sections. After deparaffinization, xylene and ethanol were used to rehydrate the slides, then we stained them with freshly prepared oil-red O working solutions for 15 min. The slides were then rinsed with 60% isopropanol, continued with 4 min of light staining with hematoxylin, then rinsing with distilled water. We mounted the slides with Entellan from Marck & Co. (Kenilworth, New Jersey, United States). Images of the slides were captured using Leica Application Suite (LEICA, Wetzlar, Germany). A total of lipid droplets from each slide was quantified using Image J, 10 fields from each sample, then averaged (33). 

***Total RNA extractions and semi-quantitative PCR***

The total RNA of the liver of male Wistar rats was extracted using TRIsure reagent (Bioline, London, United Kingdom) according to the manufacturer’s instructions. Purity and concentrations of RNA were assessed using spectrophotometry analysis, at 268/280 nm absorbance (Multiscan Go, Thermo Fisher Scientific, Massachusetts, United States). Semi-quantitative PCR was performed using The One-Step RT PCR Kit (Bioline, London, United Kingdom). The housekeeping gene GAPDH was measured for each sample as an internal control and normalization. Gel electrophoresis was performed using Mupid Exu Submarine Electrophoresis System (Mupid Co., Tokyo, Japan), while visualization of the gels was conducted using BluPAD Dual LED Blue/White Light Transilluminator (Bio-Helix Co., Taiwan). For quantification of the PCR bands using Image J (34). The list of primer sequences was provided in Table 1. 

***Statistical analysis***

All data obtained in this study were presented as mean±SEM. The results were analyzed using the Kruskal Wallis one-Way analysis of variance test, followed by LSD/Mann Whitney for the *post hoc* test. Statistical analysis was performed using SPSS software 20.0. The level significance test was fixed at *P*<0.05. 

## Results

***STZ Injection increased liver weight/body weight ratio***

**Figure 1:**
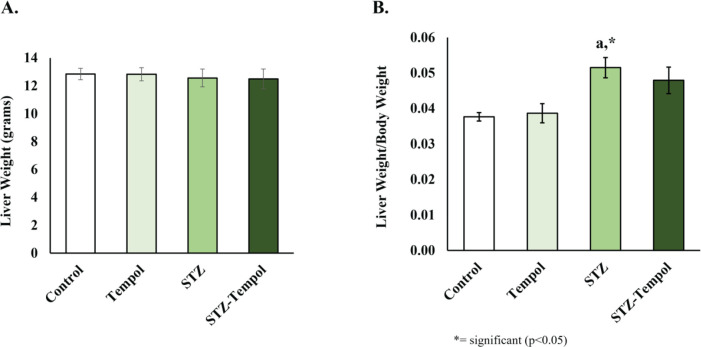
STZ injection increased liver/body weight ratio in rats. (A) No difference in liver weight between any groups. (B) Liver weight/body weight ratio increased in streptozotocin (STZ) groups compared with the control (a; *P<*0.05), but no significant differences were found in other groups Tempol: MitoTEMPOL group; STZ: Streptozotocin group; STZ-Tempol: Streptozotocin+MitoTEMPOL group

As shown in Figure 1, STZ injection did not influence liver weight (Figure 1A) but increased liver weight/body weight (Figure 1B). The significant increase in liver weight/body weight ratio between control and STZ (*P*=0.0385) was the result of decreased body weight induced by STZ injection, but no significant changes were found in other groups (Figure 1B).

***Effect of MitoTEMPOL on histopathological appearance after STZ injection***

**Figure 2:**
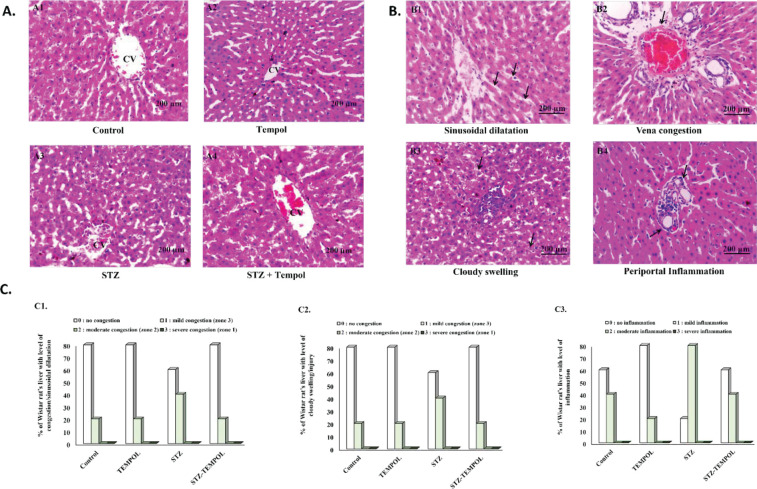
General appearance and scoring of liver histopathology after streptozotocin (STZ) injection and MitoTEMPOL ingestion (A) General appearance of liver histopathology of all groups (A1=Control, A2=TEMPOL, A3=STZ, and A4=STZ-TEMPOL). (B) Representative figures of each liver histopathology scoring (B1=Sinusoidal dilatation, B2=Vena congestion, B3=Clouding swelling, and B4=periportal inflammation). (C) Percentage of Wistar rat liver for each histopathology scoring (C1=Congestion/sinusoidal dilatation, C2=Cloudy swelling/injury, and C3=Inflammation). No significant differences in liver histopathology scoring were found between any groups

**Figure 3:**
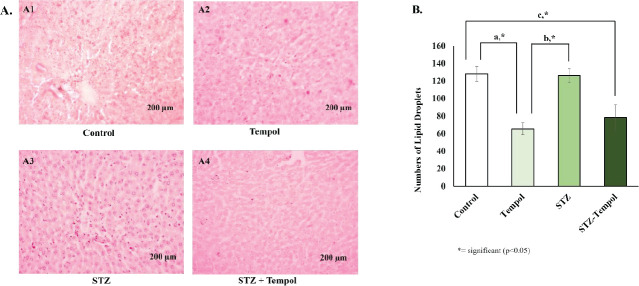
Oil red O staining of Wistar rat liver after streptozotocin (STZ) injection and MitoTEMPOL ingestion (A) Representative Figures of Oil Red O Staining from each group of the experiment (A1=Control, A2=TEMPOL, A3=STZ, and A4=STZ-TEMPOL) (B) Graphical result of numbers of lipid droplet quantification from each group of experiment. There were significant differences (*P<*0.05) between control and TEMPOL (a), TEMPOL and STZ, and between Control and STZ-TEMPOL (b)

We examined the histopathological appearance of the liver based on the scoring system applied in our previous study (33). The general histopathological appearance of all groups was presented in Figure 2A (A1=control, A2=TEMPOL, A3=STZ, A4=STZ-TEMPOL), while the representative figure of each scoring system (B1 and B2=congestion/ sinusoidal dilatation, B3=cloudy swelling/injury, B4=inflammation) was presented in Figure 2B, and graphical result to show the percentages of samples for each scoring was shown in Figure 2C. For congestion/sinusoidal dilatation, we found 80% samples with level 0 and 20% samples with level 1 in control, TEMPOL, and STZ-TEMPOL groups; but we found 60% samples with level 0 and 40% samples with level 1 in the STZ group (Figure 2C-1). For cloudy swelling/injury, we found 100% samples with level 0 in the control and TEMPOL groups, 60% samples with level 0 and 40% samples with level 1 in the STZ group, and 80% samples with level 0 and 20% samples in level 1 in STZ-TEMPOL group (Figure 2C-2). For inflammation, we found 60% samples with level 0, 40% samples with level 1 in control and STZ-TEMPOL groups, 80% samples with level 0, and 20% samples with level 1 in the TEMPOL group, 20% samples with level 0, and 80% samples with level 1 in the STZ group (Figure 2C-3). 

***Effect of MitoTEMPOL on number of lipid droplets after STZ injection***

The effect of MitoTEMPOL on a number of lipid droplets was presented in Figure 3. Figure 3A showed a representative figure of each group (Control, TEMPOL, STZ, and STZ-TEMPOL), while Figure 3B presented a graphical result for quantification of lipid droplets in each group, and we found a significant decrease of lipid droplets in Tempol and STZ-TEMPOL groups compared with control and STZ groups (*P*<0.05).

***Effect of MitoTEMPOL on mitophagy gene expression after STZ injection***

**Figure 4:**
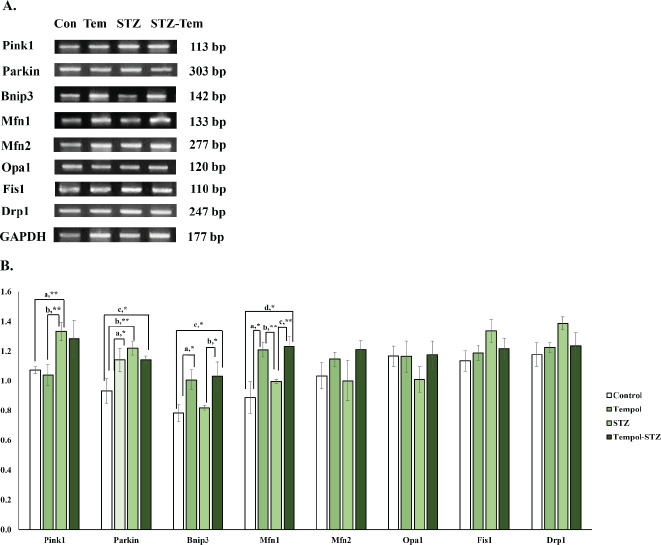
Effect of MitoTEMPOL on mitophagy gene expression after streptozotocin (STZ) injection in rat. (A) PCR bands of mitophagy gene expression after STZ injection and MitoTEMPOL ingestion. (B) Significant increase in Pink1, Parkin gene expression was found in the STZ group compared with control, but we found a decrease of BNIP3 and Mfn1 in STZ group compared with control and TEMPOL groups. But we found no significant differences in Mfn2, Opa1, Fis1, and Drp1 gene expression between any groups

We presented the bands of mitophagy gene expression (Pink1, Parkin, BNIP3, Mfn1, Mfn2, and Drp1) in Figure 4A, and the graphical result of mitophagy gene expression in Figure 4B. We found a very significant increase in PINK1 gene expression in the STZ group compared with the control (1.24-fold, *P*=0.009) and TEMPOL group (1.28-fold, *P*=0.009). We also found a significant increase in Parkin gene expression in the TEMPOL group (1.22-fold, *P*=0.033), STZ group (1.31-fold, *P*=0.005), and STZ-TEMPOL group (1.22-fold, *P*=0.034) compared with Control. For BNIP3 gene expression, we found a significant increase in the TEMPOL group compared with the control (1.28-fold, *P*=0.029), and in the STZ-TEMPOL group compared with STZ (1.26-fold, *P*=0.036) and control (1.32-fold, *P*=0.016). For Mfn1 gene expression, we found a significant increase in the TEMPOL group compared with the control (1.36-fold, *P*=0.028) and STZ group (1.21-fold, *P*=0.009); and between the STZ-TEMPOL group with STZ (1.26-fold, *P*=0.009) and control (1.23-fold, *P*=0.028). But we found no difference in Opa1 (*P*=0.515), Fis1 (*P*=0.227), and Drp1 (*P*=0.115) gene expression between any groups, as shown in Figures 4A and 4B.

***Effect of MitoTEMPOL on autophagy gene expression after STZ injection***

**Figure 5:**
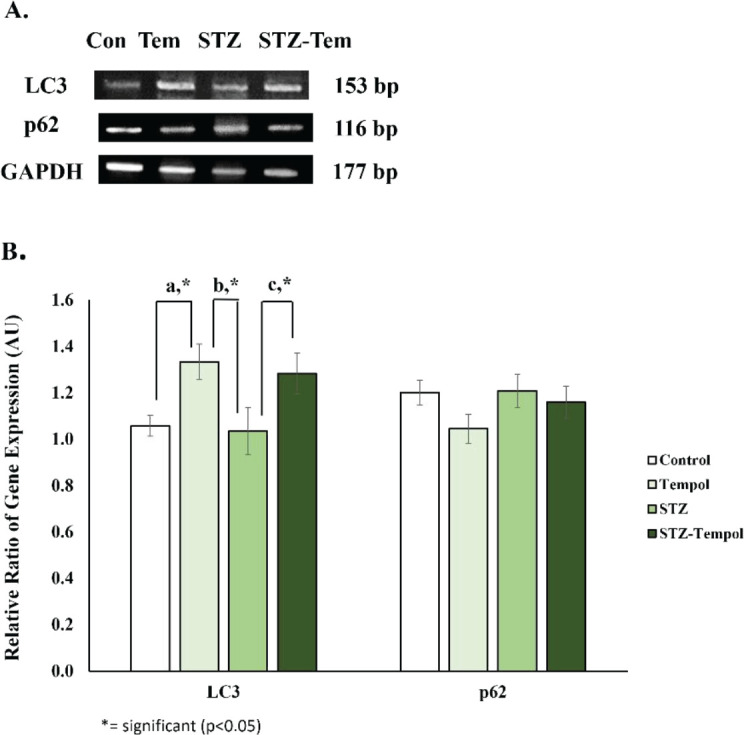
Effect of MitoTEMPOL on autophagy gene expression after streptozotocin (STZ) injection in rat. (A) PCR bands of autophagy gene expression after STZ injection and Mitotempol ingestion (B) A significant difference in LC3 gene expression was found in TEMPOL compared with control and STZ, and between STZ-TEMPOL and STZ, but no difference in p62 gene expression in any groups

We presented the bands of autophagy gene expression (LC3 and p62) in Figure 5A and the graphical result of autophagy gene expression in Figure 5B. We found no significant difference between any groups, but interestingly we found a significant increase of LC3 gene expression in the TEMPOL group compared with the control (1.26-fold, *P*=0.027) and STZ groups (1.29-fold, *P*=0.018), and in STZ-TEMPOL compared with STZ group (1.24 fold, *P*=0.044). No significant differences were found in p62 gene expression (*P*=0.293), but there is a tendency for a decrease in p62 gene expression in TEMPOL and STZ-TEMPOL groups compared with control and STZ groups. 

**Table 1:** Primers used for semi quantitative-PCR analysis of mitophagy and autophagy gene expression

**Gene symbol**	**Primer sequence (5’ to 3’)** **Upper strand: sense** **Lower strand: antisense**	**Product size (bp)**	**References**
PINK1	TGCAATGCCGCTGTGTATGA	113	(35)
	TCTGCTCCCTTTGAGACGAC		
Parkin	CCAAACCGGATGAGTGGTGAGTGC	303	(36)
	ACACGGCAGGGAGTAGCCAAGTTG		
BNIP3	GAAGCGCACAGCTACTCTCA	142	(37)
	TCCAATGTAGATCCCCAAGCC		
Mfn1	TGACTTGGACTACTCGTGCG	133	(35)
	GTGGCCATTTCTTGCTGGAC		
Mfn2	TCAGTAGCCAATCTGGACCT	277	(35)
	TCTCTTGGATGTAGGCCCCC		
Opa1	ATCATCTGCCACGGGTTGTT	120	(38)
	GAGAGCGCGTCATCATCTCA		
Fis1	AAAGAGGAGCAGCGGGATTA	110	(39)
	TGGGGCTCAGTCTGTAACAG		
Drp1	CGCTGATCCCGGTCATCAAT	247	(35)
	ACTCCATTTTCTTCTCCTGTTGT		
LC3	GGTCCAGTTGTGCCTTTATTGA	153	(40)
	GTGTGTGGGTTGTGTACGTCG		
p62	CTAGGCATCGAGGTTGACATT	116	(41)
	CTTGGCTGAGTACCACTCTTATC		
GAPDH	GTTACCAGGGCTGCCTTCTC	177	(42)
	GATGGTGATGGGTTTCCCGT		

Tempol: MitoTEMPOL group; STZ: Streptozotocin group; STZ-Tempol: Streptozotocin+MitoTEMPOL group

Tempol: MitoTEMPOL group; STZ: Streptozotocin group; STZ-Tempol: Streptozotocin+MitoTEMPOL group

Tempol: MitoTEMPOL group; STZ: Streptozotocin group; STZ-Tempol: Streptozotocin+MitoTEMPOL group; Pink1: PTEN-induced kinase 1; Parkin: Parkin RBR E3 ubiquitin-protein ligase; Bnip3: Bcl2/adenovirus E1B 19-kDa interacting protein 3; Mfn1: Mitofusin-1; Mfn3, Mitofusin-2; Opa1: Optic atrophy 1; Fis1: Mitochondrial fission 1 protein; Drp1: Dynamin-related protein 1

Tempol: MitoTEMPOL group; STZ: Streptozotocin group; STZ-Tempol: Streptozotocin+MitoTEMPOL group; LC3: Microtubule-associated protein 1A/1B-light chain 3; Sequestosome-1

## Discussion

STZ-induced diabetes in the rat model is one of the most common models to mimic acute and chronic complications of diabetes and the potential therapy to treat diabetes (4, 43). β cell destruction started 3 days after STZ administration, and reaches the peak of destruction after 2-4 weeks in rats, reducing β active cells that lead to a diabetic state (44, 45). In this study, we found a decrease in body weight in STZ and STZ-Tempol groups, compared with control and tempol groups (Figure 1A), and a significant increase in liver weight/body weight ratio between STZ and control (Figure 1C), whereas the liver weight of any group was not significantly different (Figure 1B). This condition might result from the injection of STZ which causes insulin decrease, inducing utilization of other energy sources that finally leads to weight loss (4,46). STZ injection was also reported to induce liver tumors in laboratory animals (47), but in this study, we found no difference in liver weight in any group, which means the increase in liver weight/body weight ratio was ultimately because of the decreased body weight. 

In this study, we also examined the liver histopathology scoring between all groups. The scoring was defined based on our previous study that correlated the metabolic zonation (Zones I, II, and III) with liver histopathology (congestion/sinusoidal dilatation, cloudy swelling/injury, inflammation) and autophagy (33, 48). For congestion/sinusoidal dilatation, we found an increase of samples with level 1 in the STZ group (40%) compared with samples with level 1 in control, TEMPOL, and STZ-TEMPOL groups (20%), as shown in Figure 2C-1. For cloudy swelling/injury, we also found an increase of samples with level 1 in the STZ group (40%) compared with control and TEMPOL groups (0%) and STZ-TEMPOL group (20%), as shown in Figure 2C-2. For inflammation, we found an increase of samples with level 1 in the STZ group (80%), compared with samples in the control and STZ-TEMPOL groups (40%) and samples with level 1 in the TEMPOL group (20%), as shown in Figure 2C-3. The increased level 1 of congestion/sinusoidal dilatation, cloudy swelling/injury, and inflammation in the STZ group might have resulted from the injury induced by STZ injection and might also be correlated with shifting of active metabolic zonation. We hypothesized that MitoTEMPOL might improve the histopathology appearance because of its role as a potent antioxidant (30, 49).

MitoTEMPOL also has been proven to have a protective role against NAFLD (Non-Alcoholic Fatty Liver Disease) by reducing lipid droplets in HFD-induced obese mice and inhibit foam cell formation to prevent atherosclerosis (50, 51). Recent studies have confirmed that the contact sites between lipid droplets and endoplasmic reticulum in mitochondria have a critical role in the cell metabolism of lipids. These contact sites are important for inter-organelle communication to maintain body homeostasis. In hepatocytes, the breakdown of this communication might induce lipid droplet accumulation as an early sign of NAFLD. In this study, we did not induce a high-fat diet for the experiment animals, and this might explain why the STZ group has a similar number of lipid droplets as the control group. Interestingly, we found a significant decrease of lipid droplets in the TEMPOL and STZ-TEMPOL groups (Figure 3) that might have a protective role in NAFLD prevention that might occur as a chronic complication of diabetes. 

Experimental animals induced by STZ are a useful model to determine the effect of beta-cell glucotoxicity in diabetes mellitus (52). Literature studies show that induction of experimental animals with single-dose STZ is a suitable model to study the mechanism of changes in mitochondrial structure and function that occur due to beta-cell glucotoxicity causing mitochondrial dysfunction (53). One of the things that cause mitochondrial dysfunction is the inhibition of mitophagy, which can be seen from the expression of PINK1, Parkin, and BNIP3 genes (54).

In this study, we found a significant increase of PINK1 and Parkin in STZ groups which might indicate an increase in mitochondrial fission, while BNIP3 gene expression and Mfn1 were decreased in STZ which might be correlated with mitochondrial fusion (Figure 4). This result showed an alteration of mitophagy gene expression in Wistar rat livers after STZ injection and in MitoTEMPOL ingestion compared with STZ and control groups. There are 3 pathways of mitophagy: PINK1/Parkin, BNIP/NIX, and FUNDC1, with PINK1/Parkin as a major pathway. The mitochondria life cycle reveals the interaction between mitochondrial fusion (Mfn1, Mfn2, and Opa1), mitochondrial fission (Fis1 and Drp1), and mitophagy (PINK1, Parkin, BNIP3, etc). Where mitochondrial fusion prevents the removal of mitochondria, fission produces impaired mitochondria as a target of autophagy (55). Mfn1 and Mfn2 mediate mitochondrial outer membrane fusion in mammals, while Opa1 mediates mitochondrial inner membrane fusion. Drp1 mediates mitochondrial fission that cycles between cytosol and the mitochondrial outer membrane (56). Increase of Mfn1 and Mfn2 showed an increase in mitochondrial outer membrane fusion, while the increase of PINK1, Parkin, and Drp1 showed activated mitochondrial fission. 

Mitochondrial fragmentation due to fission/fusion imbalance has often been linked to mitochondrial dysfunction and apoptosis in diabetes Mellitus (57). Mitochondrial fission and fusion balance is required for appropriate mitochondrial functions. These processes are important because of their role in maintaining mitochondrial DNA, segregating damaged mitochondria by mitophagy, distributing and moving mitochondria within the cell, and also for mitochondrial morphology regulation (58, 59). Induction of fusion and inhibition of fission by mitotempol might be correlated with its effect to maintain mitochondrial dynamics that might be disturbed because of STZ injection. The role of mitoTEMPOL to maintain homeostasis was also shown by the significant increase of LC3 gene expression in TEMPOL and STZ-TEMPOL groups, whereas in the STZ group, we found a decrease of LC3 and an increase of p62 (Figure 5). These results showed that autophagy might be activated after MitoTEMPOL ingestion, whereas STZ injection might inhibit autophagy, but further study should be conducted to support this hypothesis. 

## Conclusion

STZ injection in rats is a common model to mimic diabetes, and the purpose of this model is to evaluate the mechanism of MitoTEMPOL as a potent antioxidant for preventing complications of diabetes in the early stages. With its ability to prevent oxidative stress, which induces mitochondrial dysfunction in diabetes, the effect of mitoTEMPOL on Wistar rat liver induced by STZ injection is important to understand its mechanism in preventing acute and chronic complications of diabetes. MitoTEMPOL improves body weight, as seen in the increase of liver weight/body weight ratio, changes histopathology appearance, lipid droplets, and mitophagy gene expression after 5 weeks of STZ injection. In conclusion, mitoTEMPOL has an important role as a potent mitochondrial-targeted antioxidant to prevent complications of diabetes, especially with its correlation to mitophagy which might be correlated with histopathology improvement and reduced lipid droplets in the liver.

## Authors’ Contributions

RV, RL, and JWG designed the experiments; NN, RS, and DKJ performed experiments and collected data; RW, NA, and RL discussed the results and interpretation, JWG and HG drafted the manuscript; KL, RV, RL, and ASK supervised and managed the study; all of the authors approved the final manuscript. 

## Conflicts of Interest

The authors declared no conflicts of interest.
